# Case Report: Is it COPD? It is Fabry disease: a case in which bronchodilators were briefly used but not continued, prioritizing enzyme replacement therapy

**DOI:** 10.3389/fphar.2026.1843864

**Published:** 2026-06-09

**Authors:** Yanyan Li, Hongyi Zhu, Zijie Zhan, Ge Li, Sulan Huang, Changhui Xiao, Ying Li, Chao Zheng, Fan Huang

**Affiliations:** 1 Department of Respiratory and Critical Care Medicine, The Affiliated Changde Hospital of Xiangya School of Medicine, Central South University, Changde, Hunan, China; 2 Department of Science and Education Section, The Affiliated Changde Hospital of Xiangya School of Medicine, Central South University, Changde, Hunan, China; 3 Department of Radiology, The Affiliated Changde Hospital of Xiangya School of Medicine, Central South University, Changde, Hunan, China; 4 Department of Quality Control, The Affiliated Changde Hospital of Xiangya School of Medicine, Central South University, Changde, Hunan, China; 5 Department of Cardiology, The Affiliated Changde Hospital of Xiangya School of Medicine, Central South University, Changde, Hunan, China

**Keywords:** bronchodilators, chronic obstructive pulmonary disease, diagnostic inertia, enzyme replacement therapy, Fabry disease, mixed ventilatory dysfunction

## Abstract

**Background:**

Fabry disease, an X-linked lysosomal storage disorder, can present with mixed ventilatory dysfunction on pulmonary function testing. In patients with a smoking history, this finding may trigger a reflexive consideration of COPD and unnecessary bronchodilator use. We report a case where bronchodilators were briefly used following the diagnosis of Fabry disease, highlighting the clinical inertia that persists even after a definitive diagnosis.

**Case Presentation:**

A 39-year-old male with a 20-pack-year smoking history was diagnosed with Fabry disease through family cascade screening (α-galactosidase A activity: 4.93 nmol/L; plasma lyso-Gb3: 328.92 nmol/L; hemizygous GLA variant). Multisystem evaluation revealed mixed ventilatory dysfunction (FEV_1_/FVC 54.59%) without respiratory symptoms, left ventricular hypertrophy (septum 14–17 mm, posterior wall 15 mm), proteinuria (1.01 g/24 h), and corneal verticillata. Chest CT showed no emphysema. During hospitalization, inhaled budesonide plus ipratropium bromide (a bronchodilator) was briefly administered. However, recognizing that the ventilatory dysfunction was a manifestation of Fabry disease itself rather than COPD, the patient was discharged without a bronchodilator prescription. Enzyme replacement therapy (ERT) with agalsidase alfa (0.2 mg/kg every 2 weeks) was initiated as long-term disease-modifying treatment.

**Conclusion:**

Even after Fabry disease is confirmed, the presence of a smoking history and abnormal spirometry can trigger reflexive bronchodilator use, illustrating powerful clinical inertia. Respiratory physicians should strictly adhere to COPD diagnostic criteria. Once Fabry disease is diagnosed, ERT should be prioritized as the disease-modifying therapy, and bronchodilators, if used at all, should be reserved for symptomatic patients as an adjunct, not a substitute.

## 1 Introduction

Chronic obstructive pulmonary disease (COPD) remains a prevalent diagnosis in respiratory medicine, typically based on smoking history, characteristic symptoms, and persistent airflow limitation (Celli et al., 2022). However, not all patients with these features ultimately have COPD.

Fabry disease (OMIM 301500) is an X-linked lysosomal storage disorder caused by pathogenic variants in the *GLA* gene, leading to deficient α-galactosidase A activity and progressive accumulation of globotriaosylceramide (Gb3) and lyso-Gb3 (Arends et al., 2017; Blanco et al., 2024; Hallows et al., 2023). Respiratory system involvement has been reported in up to 61% of affected male patients and can mimic COPD (Magage et al., 2007). Despite this, diagnostic delays remain substantial; the Fabry Outcome Survey reported a mean delay of 13.7 years in male and 16.3 years in female patients (Mehta et al., 2004). The therapeutic consequence of misdiagnosis or diagnostic inertia is the potential use of bronchodilators—symptomatic interventions that do not address the underlying substrate accumulation. Consequently, disease-specific therapy (enzyme replacement therapy, ERT) may be postponed, allowing progressive organ damage (Hoffmann and Mayatepek, 2009; Schiffmann et al., 2009).

This case report describes a patient with molecularly confirmed Fabry disease in whom mixed ventilatory dysfunction triggered a reflexive consideration of COPD and brief bronchodilator use, despite the absence of respiratory symptoms. We discuss the pharmacological rationale for avoiding unnecessary long-term bronchodilator therapy and prioritizing ERT. Recent reviews have comprehensively summarized the mechanisms, diagnostic approaches, and therapeutic strategies for Fabry disease cardiomyopathy, highlighting the importance of early recognition and timely intervention (Pande et al., 2025; Chinese Society of Cardiology, 2024).

## 2 Case presentation (CARE guideline compliant)

### 2.1 Patient information

A 39-year-old man was referred for further evaluation following family cascade screening that confirmed Fabry disease. His mother had been diagnosed with Fabry disease 1 year earlier.

### 2.2 Chief complaint and history of present illness

Cascade screening revealed markedly reduced α-galactosidase A activity (4.93 nmol/L), elevated plasma lyso-Gb3 (328.92 nmol/L), and a pathogenic hemizygous GLA variant. The patient denied chronic cough, sputum production, or exertional dyspnea. Instead, he reported longstanding exercise- and heat-induced acroparesthesia and marked hypohidrosis—characteristic small fiber neuropathy symptoms.

### 2.3 Past medical and family history

Past medical history included IgA nephropathy diagnosed 3 years earlier, treated with corticosteroids, which were subsequently discontinued. Family history was notable for Fabry disease in his mother.

### 2.4 Physical examination

Physical examination revealed the following: temperature 36.4 °C, pulse 70 beats/min, respiratory rate 18 breaths/min, and blood pressure 129/76 mmHg. Lung auscultation demonstrated clear breath sounds with scattered dry rales. Cardiac examination showed a regular heart rate of 70 beats/min without murmurs. The abdomen was soft, non-tender, and without rebound tenderness. No lower extremity edema was present. Neurological examination showed mildly reduced pain and temperature sensation in the distal extremities.

### 2.5 Diagnostic assessment

Pulmonary function testing (30 January 2026) demonstrated moderate-to-severe mixed ventilatory dysfunction (Table 1). Mixed ventilatory dysfunction was defined as FEV_1_/FVC <70% (in this case, 54.59%) with forced vital capacity (FVC) < 80% of predicted (in this case, 77.48%). The predicted values were derived from the Global Lung Function Initiative (GLI) 2012 equations based on the patient’s age, height, and sex.

**TABLE 1:** Pulmonary function test results.

Parameter	Predicted	Actual	% Predicted
FVC (L)	4.55	3.53	77.48
FEV_1_ (L)	3.78	1.93	51.02
FEV_1_/FVC (%)	83.48	54.59	65.39
MMEF 75/25 (L/s)	4.36	0.96	22.03
MEF50 (L/s)	4.96	1.11	22.37
MEF25 (L/s)	2.14	0.43	20.00

High-resolution computed tomography showed no emphysema, bullae, or airway wall thickening; only scattered micronodules measuring less than 5 mm in diameter were present (Lung-RADS category 2, benign category) (Figure 1).

**FIGURE 1:**
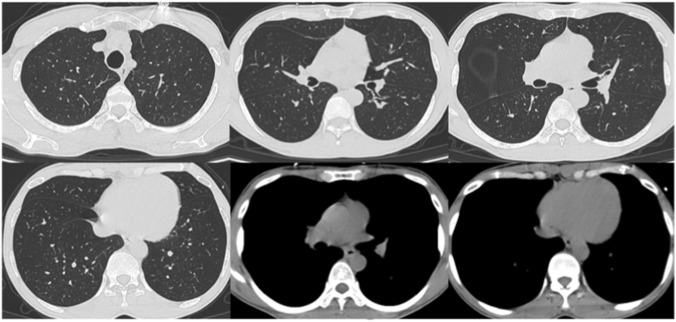
High-resolution computed tomography of the chest showing clear bronchovascular bundles with scattered micronodules (<5 mm). No emphysema or airway wall thickening is observed.

Multisystem evaluation revealed the following.

Cardiac: concentric left ventricular hypertrophy (interventricular septum 14–17 mm, posterior wall 15 mm), reduced global longitudinal strain (−12.5%), and late gadolinium enhancement on MRI (basal septum and inferolateral wall).

Renal: proteinuria (2+), 24-h urine protein 1.01 g, serum creatinine 137 μmol/L, and eGFR55.6 mL/min/1.73 m^2^.

Neurological: sympathetic skin responses showed prolonged latency and reduced amplitude, confirming small fiber neuropathy.

Ophthalmological: corneal verticillata on slit-lamp examination.

Laboratory: lipoprotein(a) 1,482.8 mg/L and myoglobin 118.97 ng/mL; other routine tests were normal.

### 2.6 Diagnostic confirmation

The diagnosis of Fabry disease was confirmed by the triad of: (1) markedly reduced α-galactosidase activity (4.93 nmol/L) (normal reference range according to the published literature: >3.8 nmol/L); (2) elevated plasma lyso-Gb3 (328.92 nmol/L) (normal reference range: <2.3 nmol/L); and (3) a hemizygous pathogenic GLA variant (Pande et al., 2025).

### 2.7 Therapeutic intervention: brief bronchodilator use and ERT initiation

During hospitalization, based on the patient’s smoking history and spirometric abnormalities, COPD was reflexively considered. The patient received inhaled budesonide plus ipratropium bromide (a short-acting anticholinergic bronchodilator) via nebulization for several days. However, after multidisciplinary review, the following observations argued against COPD: (1) absence of respiratory symptoms; (2) chest CT without emphysema; (3) multisystem involvement consistent with Fabry disease; and (4) the knowledge that mixed ventilatory dysfunction in Fabry disease is due to glycosphingolipid deposition in airway structures——a fixed, not reversible, obstruction.

Consequently, the patient was discharged without a prescription for long-term bronchodilator use. Enzyme replacement therapy with agalsidase alfa (0.2 mg/kg intravenously every 2 weeks) was initiated as disease-modifying treatment.

### 2.8 Follow-up and outcomes

After discharge, the patient has been followed monthly at our outpatient clinic. He continues the same ERT regimen (agalsidase alfa 10.5 mg every 2 weeks). As of April 2026 (approximately 2 months after initiation), he reports no worsening of symptoms and no infusion-related adverse reactions. Formal pulmonary function testing has not been repeated due to the short follow-up duration; it is planned at the 3-month visit. The patient remains adherent to ERT.

### 2.9 Patient perspective

The patient had no respiratory symptoms at diagnosis and was initially surprised that inhaled bronchodilators were briefly used. After receiving an explanation, he expressed understanding that ERT is the fundamental treatment and trusted the team’s decision to discontinue bronchodilators. Regarding smoking cessation, he acknowledged the advice but noted significant difficulty due to a long-term habit. He continues regular follow-up.

### 2.10 Diagnostic timeline

**Table:** 

Time	Event
2023	IgA nephropathy diagnosed
2025	Mother diagnosed with Fabry disease
January 2026	Cascade screening confirms Fabry disease (α-Gal A 4.93 nmol/L, lyso-Gb3 328.92 nmol/L, GLA hemizygous)
29 Jan 2026	Hospital admission
30 Jan 2026	PFT shows mixed dysfunction; chest CT without emphysema
30–31 Jan 2026	Briefly received inhaled budesonide + ipratropium bromide
February 2026	Discharged without bronchodilators; ERT initiated (agalsidase alfa 0.2 mg/kg every 2 weeks)
March–April 2026	Monthly follow-up; clinically stable, no adverse events

## 3 Discussion

### 3.1 Reflexive consideration of COPD: clinical inertia

The initial reflexive consideration of COPD was driven by the patient’s significant smoking history (20 pack-years) and the spirometric finding of mixed ventilatory dysfunction——a classic COPD pathway (Celli et al., 2022). However, several “red flags” argued against this diagnosis: the absence of typical symptoms, lack of emphysema on CT, multisystem involvement (cardiac, renal, neurological, and ophthalmological), and a positive family history. Despite the established diagnosis of Fabry disease, clinical inertia briefly led to bronchodilator use, illustrating the powerful influence of habitual diagnostic reasoning.

### 3.2 Pharmacological considerations: bronchodilators in Fabry disease

Bronchodilators (β_2_-agonists and anticholinergics) target reversible bronchial smooth muscle contraction. In Fabry disease, the primary mechanism of ventilatory dysfunction is glycosphingolipid deposition within airway smooth muscle cells, autonomic nerves, and alveolar macrophages, leading to fixed or partially fixed airflow obstruction (Magage et al., 2007; Brown et al., 1997). Electron microscopy has demonstrated lamellar inclusions (“zebra bodies”) in bronchial epithelium and smooth muscle (Franzen et al., 2013).

Published data on bronchodilator reversibility in Fabry disease are limited and mixed. Brown et al. (1997) reported positive reversibility in 5 of 8 tested patients (62.5%); importantly, all 10 methacholine challenge tests were negative in their cohort, indicating that the obstruction is not primarily due to bronchial hyperresponsiveness (i. e., there is no significant reversible component attributable to smooth muscle contraction) (Brown et al., 1997) (of note, a methacholine challenge was not performed in our patient). Most recently, Ahmed et al. (2024) reported that the prevalence of affected lung function in a national Danish Fabry cohort was 28%, with classic variants predisposing to decreased lung function independent of smoking status (Ahmed et al., 2024). Thus, even when reversibility is observed, it does not imply that bronchodilators address the underlying pathology.

European consensus guidelines recommend inhaled bronchodilators for patients with moderate or severe airway obstruction (Ortiz et al., 2018); however, the guideline explicitly states that its recommendations were not graded and were based on expert consensus rather than a systematic review. In our patient, who was completely asymptomatic from a respiratory standpoint, we considered that the potential symptomatic benefit did not outweigh the risk of unnecessary long-term medication. Therefore, bronchodilators were not continued after discharge.

### 3.3 Enzyme replacement therapy as disease-modifying treatment

ERT directly addresses the underlying enzymatic deficiency and is the only disease-modifying therapy for Fabry disease. Agalsidase alfa is a recombinant human α-galactosidase A that is taken up by cells via mannose-6-phosphate receptor-mediated endocytosis and delivered to lysosomes, where it hydrolyzes the terminal galactose residue from Gb3. The approved dose of 0.2 mg/kg every 2 weeks was established in phase III trials demonstrating stabilization of kidney function and reduction of neuropathic pain (Schiffmann et al., 2001; Eng et al., 2001). In this patient, the presence of established renal impairment (CKD stage 3a, proteinuria 1.01 g/24 h) and left ventricular hypertrophy supported prompt initiation (Schiffmann et al., 2001; Germain et al., 2015). This aligns with evidence showing that patients with less advanced disease benefit most from early treatment (Germain et al., 2015). Our patient has tolerated treatment well with no infusion-associated reactions. Although anti-drug antibodies can develop in up to 80% of male patients receiving ERT and may potentially neutralize enzyme activity, our patient has not shown any infusion-related reactions. Antibody monitoring is planned at 6 months and 12 months after ERT initiation as per routine clinical protocol.

### 3.4 Clinical lessons for respiratory physicians

This case offers several practical lessons:

1. Even after a rare disease is confirmed, clinical inertia persists. The reflexive association of smoking + abnormal spirometry with COPD briefly overrode the family history and established diagnosis of Fabry disease, leading to unnecessary bronchodilator use.
2. Strict adherence to COPD diagnostic criteria is essential. Smoking history and spirometric abnormality alone are insufficient; typical symptoms (cough, sputum, and dyspnea) and compatible imaging (emphysema or airway disease) must be present.
3. Pharmacological reasoning matters. Bronchodilators target reversible bronchospasm. Fabry disease-associated ventilatory dysfunction is predominantly structural; thus, bronchodilators are not routinely indicated. If used, they should be reserved for symptomatic patients as adjunctive support, not as primary therapy.
4. This case highlights a broader clinical principle: when encountering unexplained mixed ventilatory dysfunction—especially in patients with multisystem involvement, atypical symptoms, or a family history of genetic disorders—clinicians should broaden the differential diagnosis beyond COPD. In this particular case, the presentation of Fabry disease—a rare lysosomal disorder—with isolated pulmonary function abnormalities in the absence of respiratory symptoms provides a profound reminder that rare diseases can manifest in unexpected ways. Therefore, Fabry disease should be considered a possible etiology, and diagnostic testing (α-Gal A activity, lyso-Gb3, and GLA sequencing) should be performed when clinical clues suggest the diagnosis.

### 3.5 Limitations

Several limitations should be acknowledged. First, long-term follow-up data on ERT response are not yet available; we will report outcomes after 1 year. Second, formal bronchodilator reversibility testing was not performed during the initial hospitalization. The patient was admitted under the care of the cardiology service, and pulmonary function testing was ordered as a part of routine multisystem evaluation rather than for the purpose of diagnosing obstructive airway disease. At the time of testing, the patient was completely asymptomatic from a respiratory standpoint. Consequently, reversibility testing was not prioritized during the short hospitalization. It is planned as part of the 3-month follow-up pulmonary function assessment. Third, pulmonary function has not been reassessed after ERT initiation due to the short follow-up period. Fourth, the brief in-hospital use of bronchodilators reflects clinical inertia—we report it as a learning point, not a recommendation. Fifth, follow-up duration is too short to assess cardiac or renal response to ERT; these will be reported after 1 year of treatment.

## 4 Conclusion

This case illustrates that even after Fabry disease is confirmed, clinical inertia can trigger unnecessary bronchodilator use. Strict adherence to COPD diagnostic criteria—requiring typical symptoms and compatible imaging—could prevent such errors. Once Fabry disease is diagnosed, ERT should be prioritized as the disease-modifying therapy. Bronchodilators, if considered at all, should be reserved for symptomatic patients as supportive care, not as a substitute for ERT.

## Data Availability

The datasets generated and/or analyzed during the current study are not publicly available due to privacy or ethical restrictions. Requests to access these datasets should be directed to FH, hfan2026@163.com.
